# SARS-CoV-2 particles promote airway epithelial differentiation and ciliation

**DOI:** 10.3389/fbioe.2023.1268782

**Published:** 2023-11-02

**Authors:** Julian Gonzalez-Rubio, Vu Thuy Khanh Le-Trilling, Lea Baumann, Maria Cheremkhina, Hannah Kubiza, Anja E. Luengen, Sebastian Reuter, Christian Taube, Stephan Ruetten, Daniela Duarte Campos, Christian G. Cornelissen, Mirko Trilling, Anja Lena Thiebes

**Affiliations:** ^1^ Department of Biohybrid and Medical Textiles (BioTex), AME—Institute of Applied Medical Engineering, Helmholtz Institute, RWTH Aachen University, Aachen, Germany; ^2^ Institute for Virology, University Hospital Essen, University of Duisburg-Essen, Essen, Germany; ^3^ Department of Pulmonary Medicine, University Medical Center Essen—Ruhrlandklinik, Essen, Germany; ^4^ Institute of Pathology, Electron Microscopy Facility, RWTH Aachen University Hospital, Aachen, Germany; ^5^ Bioprinting and Tissue Engineering Group, Center for Molecular Biology of Heidelberg University (ZMBH), Heidelberg, Germany; ^6^ Clinic for Pneumology and Internal Intensive Care Medicine (Medical Clinic V), RWTH Aachen University Hospital, Aachen, Germany

**Keywords:** SARS-CoV-2, COVID-19, airway tissue engineering, lung innate immunity, epithelial cells, cilia, differentiation, stem cells

## Abstract

**Introduction:** The Severe acute respiratory syndrome coronavirus type 2 (SARS-CoV-2), which caused the coronavirus disease 2019 (COVID-19) pandemic, enters the human body via the epithelial cells of the airway tract. To trap and eject pathogens, the airway epithelium is composed of ciliated and secretory cells that produce mucus which is expelled through a process called mucociliary clearance.

**Methods:** This study examines the early stages of contact between SARS-CoV-2 particles and the respiratory epithelium, utilizing 3D airway tri-culture models exposed to ultraviolet light-irradiated virus particles. These cultures are composed of human endothelial cells and human tracheal mesenchymal cells in a fibrin hydrogel matrix covered by mucociliated human tracheal epithelial cells.

**Results:** We found that SARS-CoV-2 particles trigger a significant increase in ciliation on the epithelial surface instructed through a differentiation of club cells and basal stem cells. The contact with SARS-CoV-2 particles also provoked a loss of cell-cell tight junctions and impaired the barrier integrity. Further immunofluorescence analyses revealed an increase in FOXJ1 expression and PAK1/2 phosphorylation associated with particle-induced ciliation.

**Discussion:** An understanding of epithelial responses to virus particles may be instrumental to prevent or treat respiratory infectious diseases such as COVID-19.

## 1 Introduction

The Severe acute respiratory syndrome coronavirus 2 (SARS-CoV-2) was identified in December 2019 and rapidly progressed into the global coronavirus disease 2019 (COVID-19) pandemic (Zhu et al., 2020). Clinical manifestations of COVID-19 respiratory syndrome range from mild respiratory infections to bilateral pneumonia with acute respiratory distress syndrome (ARDS), the latter often culminating into multiple organ failure. SARS-CoV-2 uses the respiratory tract as entry gateway into the human body, where it primarily infects airway and lung epithelial cells (Jamison et al., 2022).

The airway epithelium is a pseudo-stratified cell layer mainly composed of basal, ciliated, and secretory cells, which excrete mucus. The mucus entraps potential pathogens and inhaled particles, protecting mucus-covered cells from infection (Kesimer et al., 2013). Cilia transport the mucus outwards through the respiratory tract by a process known as mucociliary clearance, ultimately leading to mucus ejection (Knowles and Boucher, 2002; Button et al., 2012). Mucociliary clearance is one of the intrinsic defense mechanisms of the lung. The relevance of mucociliary clearance becomes evident in individuals with decreased ciliation or impaired cilia motility, who are prone to severe respiratory diseases, including transmissible viruses (Legendre et al., 2021). The entry of SARS-CoV-2 into host cells is mediated by the viral spike (S) protein upon recognition of the angiotensin-converting enzyme 2 (ACE2), which is abundantly expressed by ciliated cells and presented on the cilium membrane (Hoffmann et al., 2020). A single multi-ciliated cell has around 300 cilia, multiplying the area in contact with the airway lumen by orders of magnitude. The combination of huge surface areas and high expression levels of ACE2 renders these cells into perfect entry gateways for SARS-CoV-2 (Hatmal et al., 2020; Kuek and Lee, 2020; Wu et al., 2023). Accordingly, respiratory cilia are the main target of the early SARS-CoV-2 infection (Hoffmann et al., 2020; Yao et al., 2020). Attachment to cilia is not an exclusive entry mechanism of SARS-CoV-2, but rather a common mode of action that is highly relevant for various important respiratory viruses such as influenza, rhinovirus, and the syncytial respiratory virus (Zhang et al., 2002; Griggs et al., 2017; Kuek and Lee, 2020). Accordingly, a deeper understanding of the interplay between these cells and respiratory viruses has important clinical implications.

In the trachea, ciliated cells can originate either from basal stem cells, the stem cell reservoir being located close to the basal membrane, or from club cells, also known as secretory cells, a progenitor cell type that releases protective mucus (Pardo-Saganta et al., 2015; Hogan and Tata, 2019; Fang et al., 2020; Blackburn et al., 2023). The formation of motile cilia occurs through the assembly of basal bodies and axoneme proteins, which are regulated by the transcription factor forkhead box protein J1 (FOXJ1) (You et al., 2004). The formation process is induced by the phosphorylation activity of p21-activated kinases (PAKs), and PAK1 in particular (Wu et al., 2023). In addition to mucociliary clearance, the respiratory epithelium acts as a tight barrier, protecting the body from damage and intruders. Respiratory infections often compromise the barrier integrity by destabilizing the tight junctions between epithelial cells, which are composed of occludins and claudins (Ganesan et al., 2013; Rezaee and Georas, 2014). Once the barrier is impaired, other infectious agents and/or their toxins can enter the body predisposing the host to co- and super-infections that increase the likelihood of severe diseases (Musuuza et al., 2021).

Despite the fact that SARS-CoV-2 and COVID-19 have been studied in depth, the immediate host response at the epithelium after the first contact with virus particles (called virions) is not well understood–most likely because (I) complex epithelial models such as our tri-cultures require a lot of knowledge and expertise and are not ubiquitously available, and (II) the difficulty to distinguish between cellular responses and effects caused by virus-encoded proteins and/or viral replication. For example, SARS-CoV-2 rapidly causes a global host shut-off by inducing the degradation of cellular mRNAs in the cytosol and stalling mRNA translation (Fang et al., 2020; Finkel et al., 2021; Robinot et al., 2021; Fisher et al., 2022; Wu et al., 2023), which obviously can strongly influence the respiratory epithelium. To focus on the immediate cellular response to SARS-CoV-2 virions, we exposed ciliated epithelial tri-cultures to ultraviolet (UV)-inactivated, replication incompetent SARS-CoV-2 particles. Our work sheds light on the early stages of SARS-CoV-2 virus invasion from the point of view of the airway epithelial cells.

## 2 Materials and methods

### 2.1 Cell isolation and expansion

Both human tracheal epithelial cells (HTECs) and human tracheal mesenchymal cells (HTMCs) were isolated post-mortem from lungs of organ donors at the University Medical Center Essen, Germany. Donors were selected according to the Eurotransplant guidelines. Please consult the declarations section for details regarding the ethical approval.

Cells were isolated and cultivated according to previously described methods (Mertens et al., 2017). HTECs were cultured in keratinocyte-SF medium (KSFM; Gibco) supplemented with human epidermal growth factor (EGF; Gibco, 2.5 ng/mL), bovine pituitary extract (BPE; Gibco, 25 μg/mL), isoproterenol (Sigma-Aldrich, 1 μM), and a mixture of penicillin/streptomycin (premixed, Gibco, 100 U/mL penicillin and 0.1 mg/mL streptomycin), ciprofloxacin (Fresenius Kabi, 0.01 mg/mL), and amphotericin B (PanBiotech, 2.5 μg/mL). Cells were frozen and cryo-preserved in liquid nitrogen within a mixture of 90% (v/v) KSFM, 10% (v/v) dimethyl sulfoxide (DMSO, Sigma), and BPE (0.3 mg/mL) until use. HTEC identity was regularly verified based on the expression of the basal cell marker P40/chromogranin and their capacity to differentiate into ciliated and mucus-producing cells in 2D air-liquid interface (ALI) cultures as described in Luengen *et al.* (2022). HTECs were used in P1 for differentiation in ALI conditions.

HTMCs were isolated from healthy donor tracheas at the University Medical Center Essen, Germany. The tissue was subjected to chopping and subsequent digestion with a solution of protease XIV, derived from *Streptomyces griseus* (Sigma-Aldrich, 0.6 U/mL). The HTMCs were frozen and cryo-preserved in a combination of 90% FBS and 10% DMSO. Before use, HTMCs were expanded up to passage 4 in Dulbecco’s Modified Eagle Medium (DMEM; Gibco) supplemented with 10% (v/v) fetal bovine serum (FBS; Gibco) and 1% (v/v) antibiotic/antimycotic solution (ABM; Gibco).

Human umbilical vein endothelial cells (HUVECs) were isolated from human umbilical cords by collagenase type I (Sigma-Aldrich, 400 U/mL) separation of the cell layer surrounding the vein lumen, flushing, and expanding the detached cells in endothelial cell growth medium 2 (EGM2; Promocell) supplemented with 1% (v/v) ABM. Human umbilical cords were provided by the Clinic for Gynecology and Obstetrics of the RWTH Aachen University Hospital and used within the first 24 h postpartum. HUVECs were frozen in liquid nitrogen in a freezing solution composed of 80% (v/v) DMEM, 10% (v/v) FBS, and 10% (v/v) DMSO. HUVECs were thawed and expanded up to passage 3 in EGM2 prior to the actual experiment.

### 2.2 Isolation and testing of the SARS-CoV-2 particles

The SARS-CoV-2 Delta virus was isolated from a patient sample obtained in June 2021, analyzed by Next-Generation Sequencing (data not shown) and classified with the help of GISAID and assigned into the Delta clade according to pangolin (Elbe and Buckland-Merrett, 2017; Shu and McCauley, 2017; Rambaut et al., 2020). For the isolation, Calu-3 cells (ATCC HTB-55) were incubated with virus-containing clinical nasopharyngeal swab samples until cytophatic effect was observed. The virus isolation has been approved by the ethics committee of the medical faculty of the University of Duisburg-Essen (20–9511-BO and 20–9512-BO). All work with replication-competent SARS-CoV-2 was conducted in the BSL-3 laboratory of the University Hospital Essen, Germany. For the generation of UV-inactivated viral particles (iVPs), Calu-3 cells were infected for 4 days before supernatant was collected and cleared from cells by centrifugation (500 rcf for 3 min). The supernatant containing the viral particles was treated with UV light to inactivate the virus (3 × 10 min UV light 254 nm, 3 × 10 min bench UV light). Supernatant of uninfected Calu-3 cells was also treated with UV light and served as control. iVPs were stored in aliquots at −20°C. The quantification of the viral genomes by diagnostic qRT-PCR (RealStar^®^ SARS-CoV-2 RT-PCR, Altona, Germany) resulted in a ct of 13.5 for the E gene.

The protocol for visualization of the iVPs was adapted from Le Bideau et al. (2022). Briefly, samples were centrifuged over glass coverslips at 5.000 rcf for 45 min. The iVPs were fixed in 3% glutaraldehyde (Agar scientific), rinsed with 0.1 M PBS and dehydrated in a series of ethanol dilutions with ascending concentration. The samples were then subjected to critical point drying in liquid CO_2_ and coated with a 5 nm carbon layer (Sputter Coater EM SCD500, Leica). Scanning electron microscopy (SEM) was performed in a high vacuum environment with an environmental scanning electron microscope (Quattro S ESEM, Thermo Scientific) and 10 kV acceleration voltage. The Electron Microscopy Facility of the RWTH Aachen University Hospital (Aachen, Germany) kindly supported the process.

XTT cytotoxicity assays (Cell Proliferation Kit II; Sigma-Aldrich) were carried out to assess the cytotoxicity of iVP in HTECs. HTECs were cultured until confluence in 96-well plates using airway epithelial cell growth medium (AECGM; Promocell). The iVP solution was added to cells and incubated for 24 h. As a toxic positive control, cell medium was incubated for 72 h with sheets of surgical latex gloves with a thickness of 0.13 mm in a proportion of 3 cm^2^/mL (Baek et al., 2005), while the supernatant of uninfected cells and treated in the same way as the iVP solution was used as the negative control. Subsequently, the media was removed and a mixture of XTT solution and Electron Coupling Reagent (50:1) was added to the well. Plates were incubated for another 4 h and the medium absorption was measured with an Infinite F200 Microplate Reader (TECAN) at a wavelength of 450 nm and a reference wavelength of 650 nm.

### 2.3 Preparation of the airway tri-culture models

HUVECs and HTMCs were detached by trypsin/EDTA (Pan-Biotech) digestion. Cells were then suspended in tris-buffered saline (TBS) solution containing thrombin (Sigma-Aldrich) and calcium chloride (CaCl2; Sigma-Aldrich). This mixture was combined with a fibrinogen solution (VWR) inside cell-culture inserts (PET membrane, 1.12 cm^2^ culture area, 0.4 µm pore size, Transwell^®^, Corning^®^) placed in 12-well plates (ThinCert™, Greiner Bio One). The fibrinogen was immediately cleaved by the thrombin forming a cell-loaded hydrogel with an end volume of 206 µL. The resulting gel contained 5 mg/mL fibrinogen, 3 IU/mL thrombin and 3.75 mM CaCl2 dissolved in 50 μg/mL TBS, and was loaded with 3 × 106 cells/mL of HUVECs and the same number of HTMCs. The fibrin gel was incubated for 20 min at room temperature and another 20 min at 37°C and 5% CO2 in a humidified atmosphere to achieve the complete polymerization of fibrin. HTECs were then thawed and directly seeded on top of the hydrogels in a cell density of 80,000 cells/cm2. These tri-culture models were then cultured under submerged conditions for a week in proliferation media, composed of a 1:1 mixture of EGM2 and AECGM supplemented with 40 μg/mL gentamicin (Rotexmedica) and 0.16 mg/mL tranexamic acid (TXA; Carinopharm) to avoid degradation of the gels by fibrinolysis (Cholewinski et al., 2009). Subsequently, gels were switched to ALI conditions by adding differentiation medium only to the basal side and leaving the epithelialized surface exposed to the air for 4 weeks, to achieve a full maturation of the cell layer. The differentiation medium was a 1:1 combination of EGM2 and MucilAir (Epithelix) supplemented with 20 μg/mL gentamicin and 0.16 mg/mL TXA. Cell culture media were exchanged every 2–3 days. All tri-cultures were prepared with cells derived from the same HUVEC and HTMC donors, but varying HTEC donors, to take inter-individual variations into account.

For assessing the SARS-CoV-2 permissiveness of fully matured tri-cultures, they were inoculated with replication competent SARS-CoV-2 and fixed 8 days post-infection for immunofluorescence staining of the SARS-CoV-2 nucleocapsid (N) protein.

For the experiments with the inactivated SARS-CoV-2, tri-cultures were treated with 100 µL of iVP from SARS-CoV-2 Delta strain. Samples were fixed for SEM and histology 4, 24, and 48 h post-treatment. Controls were not treated with iVP but directly fixed.

### 2.4 Quantification of the ciliated surface area by scanning electron microscopy

The epithelium surface of the tri-culture models was imaged by SEM. Samples were fixed in 3% (v/v) glutaraldehyde, rinsed with PBS, and dehydrated using ethanol solutions with ascending solvent content. Samples were then subjected to critical point drying in liquid CO_2_, and coated with a 10 nm gold/palladium layer (Sputter Coater EM SCD500, Leica). SEM was performed in a high vacuum environment with environmental scanning electron microscopes (either XL30 ESEM FEG, Philips, or Quattro S ESEM, Thermo Scientific) and 10 kV acceleration voltage.

The percentage of the ciliated surface area visualized by SEM images was evaluated by ten trained observers, who did not receive information regarding the experimental conditions. The evaluators classified the images into five categories concerning the total ciliated area: (I) less than 20%, (II) between 20% and 40%, (III) between 40% and 60%, (IV) between 60% and 80%, and (V) more than 80% of are ciliated. Mean values were used for further calculations.

### 2.5 Histological and immunofluorescence evaluation

Samples were assessed by periodic acid Schiff’s reaction (PAS reaction) and immunofluorescence. Samples were fixed in Carnoy’s solution (6:3:1 mixture of ethanol, chloroform, and glacial acetic acid, respectively) for 1 hour, embedded in 2% (w/v) agarose and dehydrated in diluted ethanol in ascending concentrations and xylene. Dehydrated samples were embedded in paraffin and cut into 3 µm-thick sections using a microtome (pfm medical). These sections were deparaffinized with a reverse series of solutions with descending xylene and ethanol concentrations.

For the PAS reaction, samples were oxidized with 1% (w/v) periodic acid solution (Merck) for 10 min, cleared with tap water, and treated with Schiff’s reagent (Merck) for another 10 min. The slides were then incubated in tap water between 35°C and 50°C for 10 min and counterstained with hematoxylin solution (Sigma-Aldrich). Samples were dehydrated in a series of ethanol and xylene dilutions with ascending concentrations, and mounted with coverslips using Euparal (Carl Roth). Slides were imaged using an Axio Imager D brightfield microscope (Carl Zeiss).

For immunofluorescence staining, samples were first blocked with a solution of 1% (w/v) bovine serum albumin (BSA; Sigma-Aldrich) and 0.1% (w/v) sodium azide (Sigma-Aldrich) for 1 hour and then incubated in the respective primary antibody over-night at 4°C, namely, rabbit anti-pan-cytokeratin, rabbit anti-claudin-1, mouse anti-acetylated tubulin, rabbit anti-FOXJ1, mouse anti-TP63, rabbit anti-CCSP, goat anti-ACE2, mouse anti-SARS-CoV-2 nucleocapside, and rabbit anti-pPAK1/2 (Supplementary Table S1). Samples were washed, treated with the secondary antibody for 1 hour at 37°C, washed, stained with 0.4 μg/mL of a DAPI solution, washed again, and covered with a coverslip using fluorescent mounting medium (Dako). Slides were imaged using an inverted Axio Observer Z1 fluorescence microscope (Carl Zeiss) or a Nikon Eclipse TI (Oko Lab).

### 2.6 Statistical analysis

All statistical analyses were performed using GraphPad Prism 9.3.1. Ciliated area evaluations and XTT assay results were checked for normality with the Shapiro-Wilk test. One-way analysis of variance (ANOVA) with Tukey’s *post hoc* test was performed to compare all conditions.

## 3 Results

### 3.1 Airway tri-cultures are a suitable model for the early stages of SARS-CoV-2 infection

**FIGURE 1:**
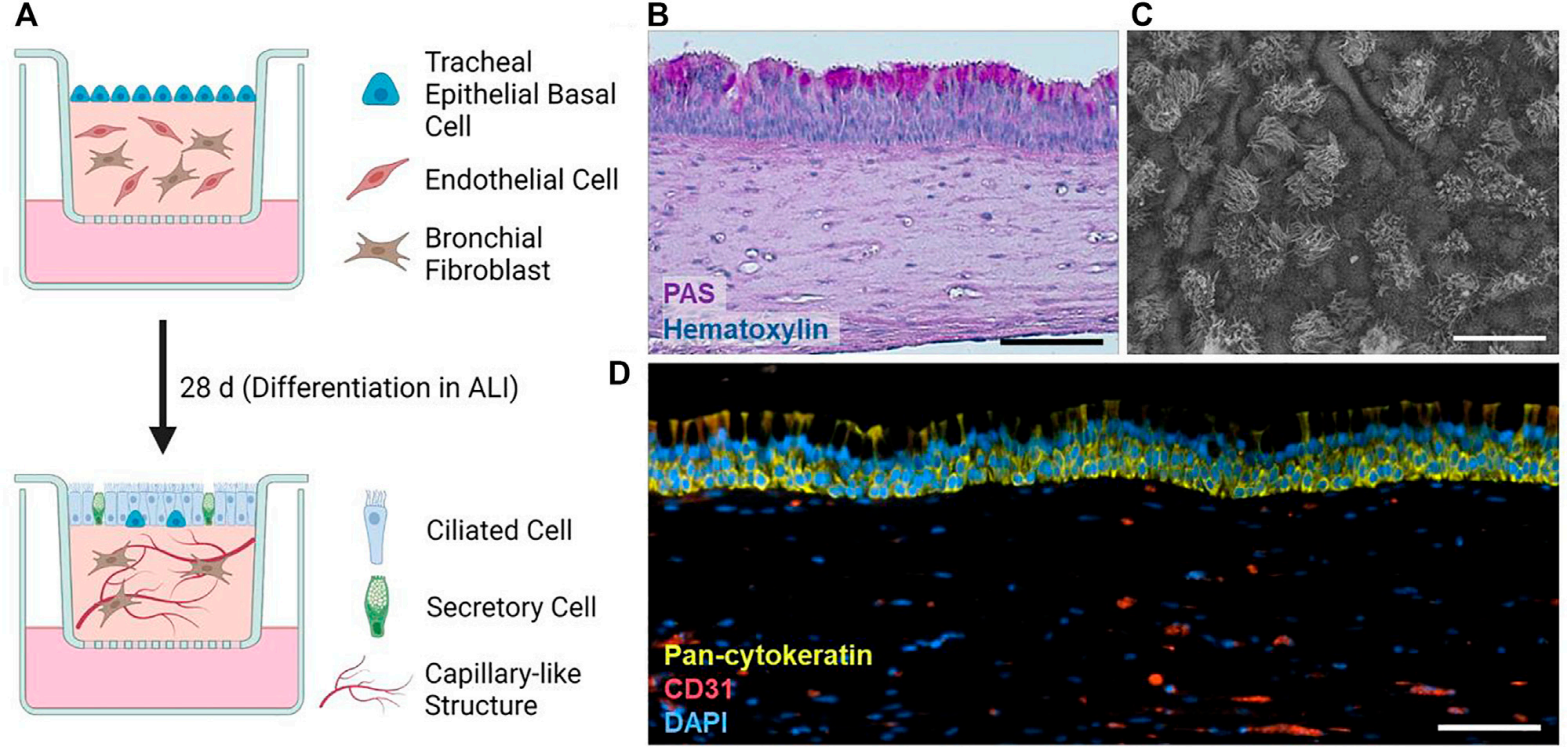
**(A)** Schematic overview of the tri-culture airway model and its differentiation. Created with BioRender. **(B)** PAS reaction of a histological section of a typical tri-culture model. **(C)** Prototypic SEM image of the differentiated tri-culture model epithelium. **(D)** Typical immunofluorescence staining of pan-cytokeratin (green), cell nuclei (DAPI), and CD31 (red) within tri-culture models. Scale bars: 100 µm **(B,D)**, 20 µm **(C)**.

Our airway tri-cultures consist of endothelial cells and human tracheal mesenchymal cells in a molded fibrin hydrogel covered by a layer of tracheal epithelial cells (Figure 1A). Gels were cultured for 1 week submerged in proliferation medium, and subsequently for 4 weeks in ALI mode with differentiation medium. During maturation, HTECs form a pseudo-stratified layer comprising both basal and columnar cells, including secretory cells, which release mucus to the surface, as evident by the PAS reaction (Figure 1B). Furthermore, SEM imaging (Figure 1C) confirmed the presence of ciliated cells in the epithelium, and immunofluorescence staining recognizing CD31 and pan-cytokeratin markers showed the location of endothelial and epithelial cells, respectively (Figure 1D).

**FIGURE 2:**
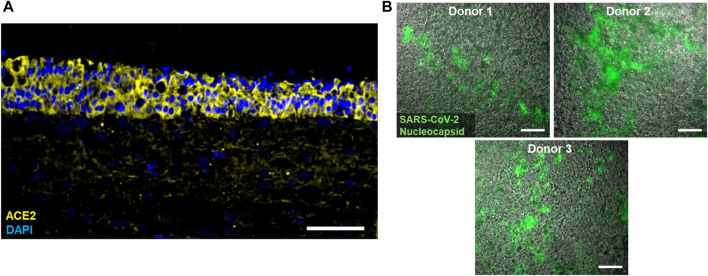
**(A)** Typical immunofluorescence staining of cell nuclei (DAPI; blue) and the ACE2 receptor (yellow) in tri-culture models. **(B)** Immunofluorescence staining of the SARS-CoV-2 Nucleocapsid (green) in tri-culture models from three different epithelial donors 8 days post infection with replication competent SARS-CoV-2. Scale bars: 100 µm **(A)**, 20 µm **(B)**.

To analyze the potential to serve as a model for SARS-CoV-2 infection, immunofluorescence stainings of the ACE2 receptor were performed, which indicated that the epithelium expresses high levels of ACE2 (Figure 2A). Accordingly, and consistent with our findings in the context of other projects (Schuhenn et al., 2022) SARS-CoV-2 multiplied in the cultures as evident by the formation of nucleocapsid protein (N)-positive plaques 8 days post infection (dpi) with replication competent SARS-CoV-2 (Figure 2B).

### 3.2 Exposition of the airway tri-culture to inactivated SARS-CoV-2 particles increases ciliation

To interrogate which immediate consequences the contact between SARS-CoV-2 with respiratory tissues has, we decided to minimize the influence of viral gene expression and/or cell damage caused by viral replication by inactivating the virus. To this end, we used UV light irradiation, which efficiently abrogates the replication competence of SARS-CoV-2 (Heilingloh et al., 2020). After UV irradiation, SEM imaging of the inactivated virus particles (iVP) showed the expected presence of round viral particles with diameters between 100 and 150 nm (Supplementary Figure S1A), suggesting that the UV light does not compromise the overall integrity of virions, but rather acts through damage of the genomic RNA. Accordingly, cytotoxicity assays performed in HTECs mono-cultures showed that the iVP do not elicit detectable cytotoxicity in comparison to the negative control after 24 h of incubation, while the positive control (latex-conditioned media) showed a significant decrease of cell viability (Supplementary Figure S1B).

**FIGURE 3:**
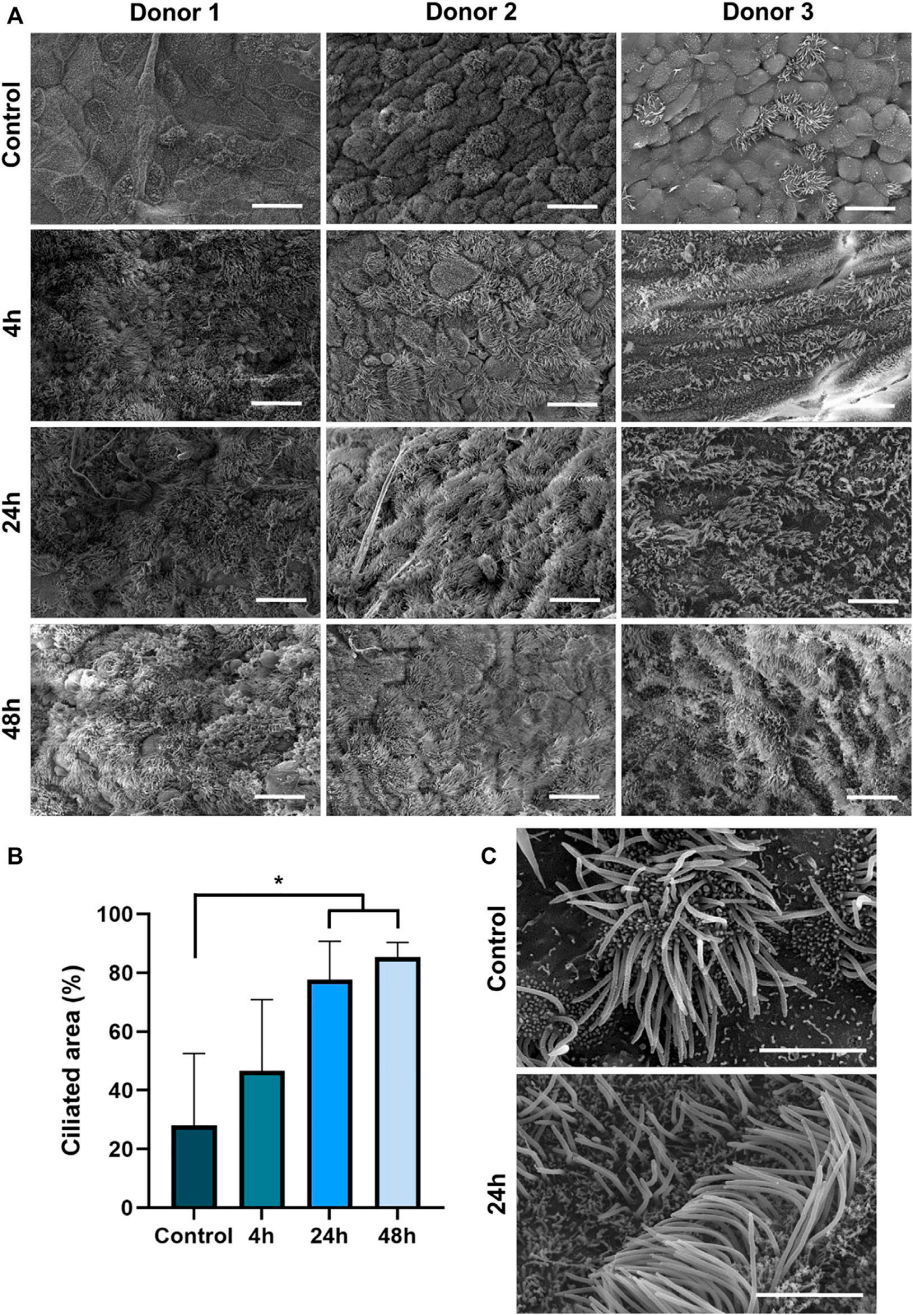
**(A)** SEM imaging of the tri-culture model before (control) and 4, 24 or 48 h after inoculation with SARS-CoV-2 iVP. **(B)** Median ciliated surface area in tri-culture models after inoculation with iVP as judged by ten trained observers (n = 3 donors, using ANOVA, *p < 0.05). **(C)** Detailed SEM images of multiciliated epithelial cells in an untreated tri-culture and 24 h post iVP inoculation. Scale bars: 20 µm **(A)**, 4 µm **(C)**.

Intriguingly, SEM imaging revealed an astonishing trans-differentiation of non-ciliated HTECs of the tri-culture airway surface into ciliated cells after contact iVP (Figure 3A). This process was already detected 4 h after start of the exposure and increased over time. Quantification of the ciliated area revealed statistically significant differences 24 and 48 h post-inoculation compared to the control (Figure 3B). The augmented SEM images show that the cilia in the control and 24 h after the exposure to the iVPs are phenotypically indistinguishable from each other (Figure 3C), presenting similar diameters and lengths.

### 3.3 SARS-CoV-2 particles provoke a decrease in CCSP, TP63, and claudin-1 positivity

**FIGURE 4:**
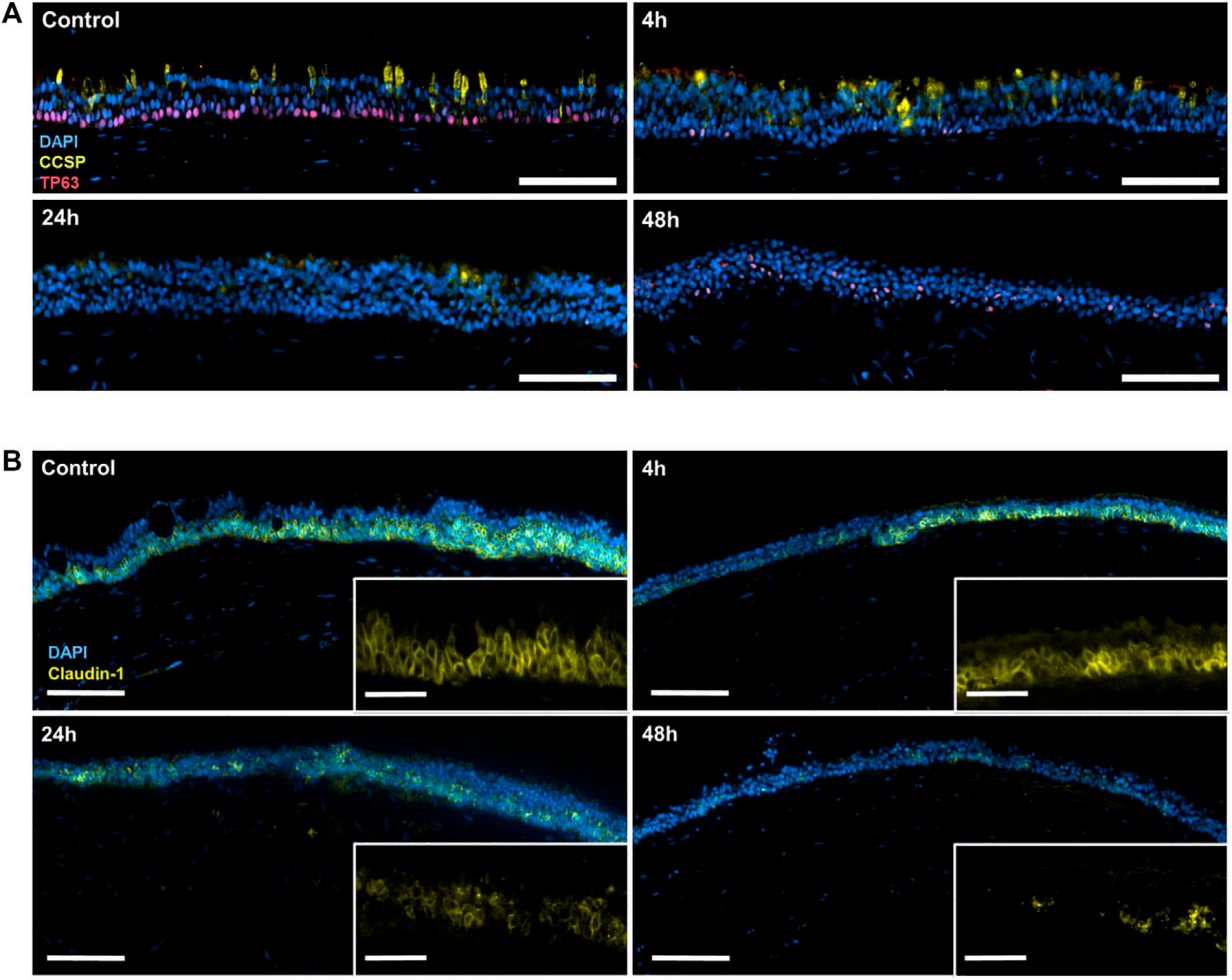
**(A)** Immunofluorescence staining of cell nuclei (DAPI; blue), CCSP (yellow) and TP63 (red) in control tri-culture models or 4, 24, and 48 h after inoculation with the iVP. **(B)** Immunofluorescence staining of cell nuclei (DAPI; blue), claudin-1 (magenta) and acetylated tubulin (yellow) in the control tri-culture model and 4, 24 and 48 h after inoculation with SARS-CoV-2 iVP. Scale bars: A, 100 μm; B, 100 µm (overview) and 20 µm (magnified).

Tumor protein 63 (TP63) is a key regulator of epithelial proliferation and differentiation, specifically expressed in the basal stem cells of the airway (Hogan et al., 2014). The immunofluorescence staining of TP63 revealed the presence of basal stem cells in the lower part of the pseudo-stratified layer before the inoculation with the iVP, followed by a drastic expression reduction directly after exposure (Figure 4A). In addition to a reduction in numbers, the TP63+ cells also appear in a higher position within the layer 48 h after the exposure. Similarly, stainings of the club cell secretory protein (CCSP, also known as Uteroglobin or secretoglobin family 1A member 1 [SCGB1A1]) showed the presence of these secretory cells in the control, but the signal started to decline 4 h after the exposure and eventually disappeared 24 and 48 h after start of the exposure.

Furthermore, the immunofluorescence staining of claudin-1, one of the main cell-cell tight junction proteins in the epithelium, demonstrated the intercellular connection between HTECs before the exposure to iVP (Figure 4B). However, this staining progressively decreased after the iVP exposure.

### 3.4 FOXJ1 induction and PAK1 activation precede the iVP-induced ciliation

**FIGURE 5:**
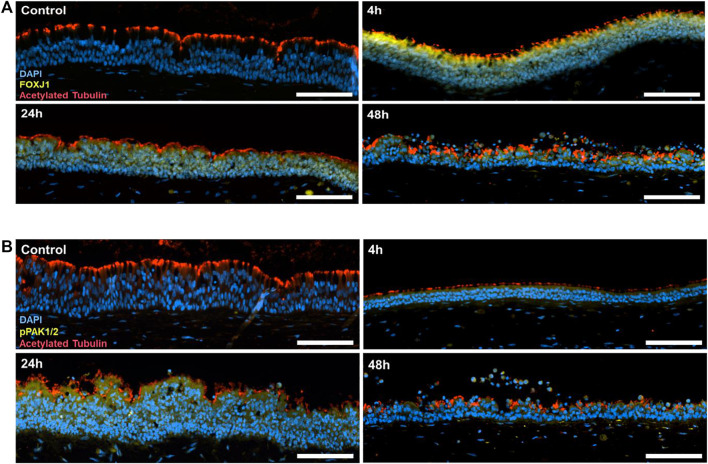
**(A)** Immunofluorescence staining of cell nuclei (DAPI; blue), the FOXJ1 transcription factor (yellow) and acetylated tubulin (red) in the tri-culture model in the control cultures, and 4, 24 or 48 h after inoculation with SARS-CoV-2 iVP. **(B)** Immunofluorescence staining of cell nuclei (DAPI; blue), pPAK1/2 (yellow) and acetylated tubulin (red) in the tri-culture model and 4, 24 and 48 h after inoculation with SARS-CoV-2 iVP. Scale bars: 100 µm.

The results of the immunofluorescence staining showed no expression of the FOXJ1 transcription factor prior to iVP exposure. Conversely, we observed an intense increase after 4 h of contact with iVP (Figure 5A), indicating a rapid FOXJ1 induction in response to SARS-CoV-2 particles. However, there was a slight decrease in the expression of FOXJ1 at 24 and 48 h post-exposure, indicating the initial response to the virus as temporary. In addition, the PAK1/2 kinases were activated by phosphorylation after exposure to the iVP (Figure 5B). This activation was particularly pronounced 24 h but less obvious 4 and 48 h after the inoculation, showing a time-dependent response of the respiratory epithelium to SARS-CoV-2 particles. Cilia presence over all the epithelium surface is confirmed by the acetylated tubulin staining, not showing substantial differences between the different time points.

## 4 Discussion

Various groups have convincingly shown that SARS-CoV-2 infects ciliated respiratory epithelial cells *in vitro* and *in vivo* (Fang et al., 2020; Robinot et al., 2021; Wu et al., 2023). This infection is consistent with their localization at the interface between the body and the airways through which inhaled virus-containing droplets traverse, the high surface area, and the abundant expression of the entry receptor ACE2. Accordingly, ciliated cells are the major cellular target of the SARS-CoV-2 infection in the airway (Robinot et al., 2021; Jamison et al., 2022). In agreement with the SARS-CoV-2 permissiveness and the cytopathic nature of the virus, replicating SARS-CoV-2 reduces the number of ciliated cells over time (Robinot et al., 2021). Thereby, COVID-19 provokes the destruction of ciliated cells and leads to a reduction in mucociliary clearance, which in turn promotes the accumulation of mucus and debris in the airway, providing an optimal environment for the virus to replicate and spread (Fang et al., 2020). Moreover, the loss of ciliated cells can also impair the barrier function of the airway epithelium, rendering the host more susceptible to secondary bacterial infections (Ganesan et al., 2013; Rezaee and Georas, 2014). Importantly, we do not question these facts. However, it is crucial to emphasize that aforementioned experiments which showed the loss of ciliated cells were conducted with replicating SARS-CoV-2 which was given the opportunity to undergo multiple rounds of multiplication. In clear contrast, we provide evidence that inactivated SARS-CoV-2 particles by nature stimulate an enrichment of cilia. The fact that others also observed this immediate ciliation response to SARS-CoV-2 particles rules out that this is an artefact (Wu et al., 2023). Thus, the questions are: (I) which viral determinants induce the ciliation, (II) by interacting with which cellular factors and (III) why? One main novelty of our study is the finding that UV-irradiated SARS-CoV-2 particles are sufficient to induce this ciliation response. The fact that our iVP were strongly UV-irradiated suggests that particles are not only replication incompetent but also inert in terms of transcription. Accordingly, we hypothesize that the viral ciliation-inducing factor encoded by SARS-CoV-2 is sufficiently abundant in the virion. SARS-CoV-2 contains four main structural proteins: spike (S), nucleocapsid (N), membrane (M), and envelope (E). Due to the following reasons, E might be a potential candidate for the observed effects on tight junctions and ciliation: The c-terminus of E contains a PDZ-domain binding domain (‘DLLV’) that interacts with the tight junction protein ZO1 and PALS1 (Caillet-Saguy et al., 2021; Chai et al., 2021; Shepley-McTaggart et al., 2021). Intriguingly, the conditional ZO1 deletion in intestinal epithelial cells results in striking morphologic abnormalities that include disruption of apical surface architecture and the induction of protrusions (Odenwald et al., 2018).

The rapid increase in ciliation observed in our study could also represent a compensatory mechanism to counteract the loss of ciliated cells induced by the virus and boost the mucociliary clearance, accelerating the expulsion of viral particles out of the respiratory system. The mechanism appears to be connected to the increase in the FOXJ1 transcription factor within the first 4 h after exposition to the iVP. FOXJ1 is a protein required for the formation of motile cilia by basal body docking and axoneme growth (You et al., 2004; Jain et al., 2010). The expression of FOXJ1 decreased again after most of the epithelial cells have already become ciliated, at 24 and 48 h. Robinot et al. (2021) detected downregulation of FOXJ1 at 2 days post-inoculation, followed by impairment of the ciliary function. However, here–to our knowledge for the first time–FOXJ1 expression is studied in the first hours upon contact with SARS-CoV-2 iVP, suggesting that the early cellular response is a transient increase of FOXJ1 expression. FOXJ1 induction is required for the generation of ciliated cells from basal stem cells and club cells, through the activation of the STAT3 transcription factor (Tadokoro et al., 2014; Tanabe and Rubin, 2016; Kameyama et al., 2017). Robinot et al. (2021) showed that respiratory epithelial cells synthesize both interleukin 6 (IL 6) and interferon-γ soon after SARS-CoV-2 inoculation as an antiviral defense. Both cytokines are known to activate the expression of STAT3 (van Wissen et al., 2002; Zhu Y. et al., 2019), raising the question if interferons and/or IL-6 are involved in SARS-CoV-2-induced ciliation.

The cytoskeletal organization and transportation necessary for the formation of new cilia are mediated by the PAK family of kinases. The activation of PAK1/2 by phosphorylation that could be observed after the iVP exposition was probably related to the basal body and axoneme protein trafficking necessary for the rapid ciliation. The same phenomenon was reported by Wu et al. (2023) in the nasal epithelium, where PAK phosphorylation was involved in the microvilli elongation that aid the SARS-CoV-2 egress and spread.

The immunohistological stainings suggest that the club cells and/or the basal stem cells are the precursors of the newly formed ciliated cells. The club cells are an epithelial population present along the airway which secrete CCSP as a protectant and act by detoxifying harmful substances in contact with the respiratory lumen (Rokicki et al., 2016; Blackburn et al., 2023). These cells also act as progenitors giving rise to either ciliated or goblet cells (Hogan et al., 2014; Pardo-Saganta et al., 2015; Hogan and Tata, 2019). The presence of CCSP expression was maintained 4 h after exposure to iVP, but it decreased significantly in the next 20 h, suggesting the differentiation of the club cells into ciliated cells in response to the viral proteins.

At the same time, a strong fading of TP63-positive cells was observed after the addition of the iVP, with some TP63 signal appearing in the upper layers of the epithelium instead of the basal area, indicating a suprabasal migration during the generation of early progenitor ciliated cells. TP63 is a member of the p53 family of transcription factors and is known to be a key regulator of stem cell self-renewal and differentiation in the airway epithelium, with its expression being largely restricted to basal stem cells (Warner et al., 2013; Post and Clevers, 2019; Bilodeau et al., 2021). TP63 expression is known to be downregulated as the basal cells lose their stem cell identity and differentiate towards a ciliated cell fate (Pardo-Saganta et al., 2015).

It is not clear, however, if these morphological changes were innocuous to the epithelium barrier integrity. Healthy airway epithelial cells are attached to each other forming a compact layer, with tight junction complexes binding the epithelial lateral membranes, where claudins are one of the major components. Airway barrier integrity is compromised in disease leading pathogens to enter more easily the submucosa and reach the inner tissues (Ganesan et al., 2013; Kojima et al., 2013; Rezaee and Georas, 2014). Here, a time-dependent decrease of the claudin-1 signal between the epithelial cells was observed, pointing to a possible alteration of the barrier function. The absence of barrier integrity analysis such as trans-epithelial electrical resistance measurements or permeability assays makes it impossible to verify an impairment of the epithelium’s integrity. However, a weakening of the epithelial barrier has already been reported in studies using the active SARS-CoV-2 virus (Robinot et al., 2021). The present study shows a disruptive effect in the claudin-1 intercellular distribution without any cytopathic effect of the iVP, thus probably attributable to the ciliation response and the subsequent transient remodeling of the epithelium. This suggests that the observed morphological changes in the airway might have consequences for the barrier integrity, supporting the hypothesis that the remodeling of the epithelium to improve mucociliary clearance may be counterproductive, facilitating the passage of the virus through the mucosa.

The damage to the barrier tight junctions is not the only phenomenon that can convert this ciliation response into a double-edged mechanism pernicious to epithelium homeostasis. Such an increase in the cellular surface susceptible to infection could aid the virus to spread and replicate more efficiently in the respiratory epithelium, potentially leading to further damage and exacerbation of the infection (Su et al., 2023). Additionally, the increase of ciliated cells may negatively influence the balance between different cell types in the respiratory epithelium which could impair its overall function and compromise the barrier against pathogen invasion (Hewitt and Lloyd, 2021). The decrease of club cells provoked by their differentiation into ciliated cells may lead to a reduction in the secreted proteins that moist and protect the epithelium barrier, a deficiency known to exacerbate other airway pathologies (Zhu L. et al., 2019; Martinu et al., 2023).

Further studies are necessary to elucidate the long-term effects of this compensatory mechanism and to understand its potential role in the pathogenesis of respiratory infections. The tri-culture model used in this research is limited by the lack of immune cells such as residence macrophages and circulatory neutrophils that also have an important role in the first stages of the response against viral infection (Hewitt and Lloyd, 2021). The current study does not report results on the effect of iVP exposure on vascularization, since the rationale behind the inclusion of endothelial cells in the hydrogel layer imitating the airway submucosa was to increase the model’s biomimicry and epithelium support (Burkhanova et al., 2022; Luengen et al., 2022). Further experiments with longer incubation times and more suited models would be needed to extract reliable information about the vessel response against the iVP presence. A limitation of this study is the lack of *in vivo* investigation of the described processes. An experiment that requires screening a precise area of the airway after contact with a known specific quantity of viral particles, and in such a short period, would be highly difficult to replicate in animal studies. However, *in vivo* application of iVPs is necessary to contextualize the recent findings as other relevant agents in the airway response such as tissue-resident and circulating immune cells are expected to be involved in the response to iVPs, too. To our knowledge, current literature lacks *in vivo* studies on the short-term effect of the inhalation of inactivated SARS-CoV-2 viral particles that could confirm or refute the present findings.

Moreover, it is important to determine whether interventions aimed at manipulating the ciliary density and function could be a viable strategy for the prevention and treatment of respiratory viral infections. These findings have also significant implications for the treatment of respiratory diseases that involve the loss or dysfunction of cilia, such as primary ciliary dyskinesia (Lucas et al., 2020; Paff et al., 2021). The ability to stimulate the regeneration of cilia in the respiratory epithelium could potentially be a game-changer in the treatment of these conditions. However, accelerated plasticity could also be detrimental to the epithelium homeostasis, leading to further damage and exacerbation of infections. As such, pro-ciliation therapies should also aim to preserve the integrity of the epithelium barrier during remodeling.

## 5 Conclusion

In conclusion, the airway tri-culture model used in this study has been proven as a valuable 3D model of the human airway with respiratory epithelial cells expressing ACE2 and which is capable to be infected with SARS-CoV-2, making it a valuable tool for studying the pathophysiology of the viral infection. The exposition of the model to previously inactivated viral particles provoked a significant increase in the presence of multiciliated cells, presumably through increased expression of the transcription factor FOXJ1, leading to the differentiation of basal stem cells and club cells. The cellular remodeling is associated with the phosphorylation of the PAK1/2 proteins and has an observable effect in the epithelial barrier tight junctions. The rapid plasticity of the respiratory epithelium observed in this study could be triggered by the SARS-CoV-2 structural components such as the envelope protein. Although mucociliary clearance may be enhanced by increased ciliation, it also increases the number of cells and surface area susceptible to SARS-CoV-2 infection and might lead to negative effects on the epithelial barrier function. A limitation of this study is that it focusses only on the early stages of SARS-CoV-2 virus invasion in airway epithelial cells. Further studies in animal models can give a more comprehensive view on processes taking place in the *in vivo* setting.

## Data Availability

The original contributions presented in the study are included in the article/Supplementary Material, further inquiries can be directed to the corresponding author.

## Abbreviations

ABM, antibiotic/antimycotic; ACE2, angiotensin-converting enzyme 2; AECGM, airway epithelial cell growth medium; ARDS, acute respiratory distress syndrome; ALI, air-liquid interface; ANOVA, analysis of variance; BPE, bovine pituitary extract; CCSP, club cell secretory protein; COVID-19, coronavirus disease 2019; DMEM, Dulbecco’s Modified Eagle Medium; DMSO, Dimethyl sulfoxide; EGF, epidermal growth factor; EGM2, endothelial cell growth medium 2; EMM, EGM2-MucilAir-Mixture; FCS, fetal calf serum; FOXJ1, transcription factor forkhead box protein J1; HTECs, human tracheal epithelial cells; HTMCs, human tracheal mesenchymal cells; HUVECs , human umbilical vein endothelial cells; IL6, interleukin-6; iVPs, UV-inactivated viral particles; KSFM, keratinocyte-SF medium; mAir, modified airway epithelial cell growth medium; NGS, normal goat serum; PAK, p21-activated kinases; PAS reaction, periodic acid-Schiff’s reaction; S, spike protein; SEM SARS-CoV-2, scanning electron microscopy severe acute respiratory syndrome coronavirus type 2; TPLSM, two-photon laser scanning microscopy; UV, ultraviolet.
