# Supplementary material for: SARS-CoV-2 particles promote airway epithelial differentiation and ciliation
Source: Front Bioeng Biotechnol. 2023 Nov 2;11:1268782. doi: 10.3389/fbioe.2023.1268782 (PMC10654538; doi:10.3389/fbioe.2023.1268782)
Supplement: Supplementary file 1 [file DataSheet1.docx]

Supplementary Material

SARS-CoV-2 particles promote airway epithelial differentiation and ciliation

Julian Gonzalez-Rubio, Vu Thuy Khanh Le-Trilling, Lea Baumann, Maria Cheremkhina, Hannah Kubiza, Anja E. Luengen, Sebastian Reuter, Christian Taube, Stephan Ruetten, Daniela Duarte Campos, Christian G. Cornelissen, Mirko Trilling, Anja Lena Thiebes*

*** Correspondence:** Anja Lena Thiebes: thiebes@ame.rwth-aachen.de

# Supplementary Figures

**
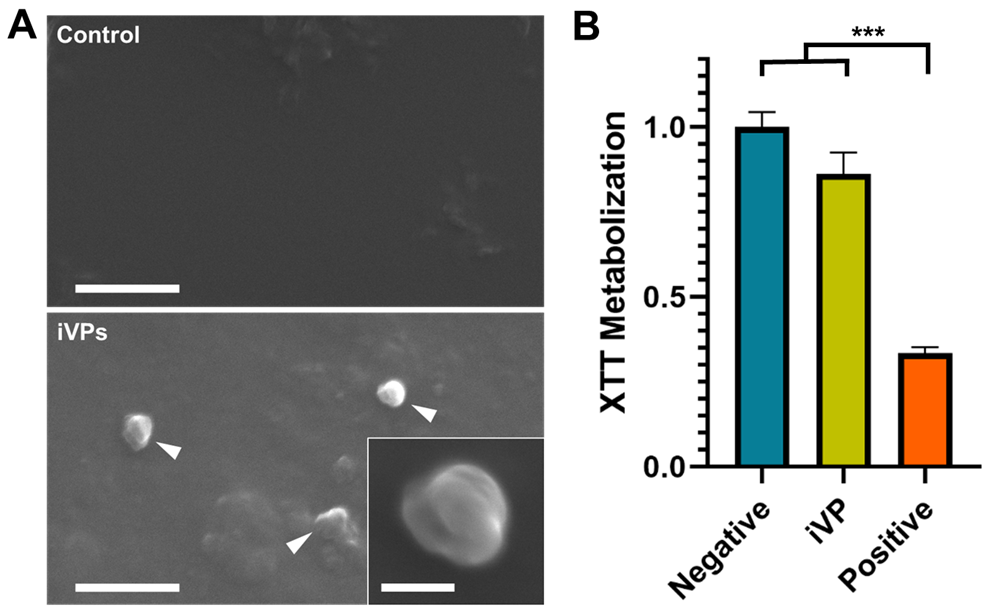
**

**Supplementary Figure S1.** (A) Prototypic SEM image of iVP. (B) Cytotoxicity assay results of the HTECs treated for 24h with SARS-CoV-2 iVP (n = 3 donors with 5 replicates each, using ANOVA, *** p < 0.0001). Scale bars: 500 nm (Overview), 100 nm (Magnified).

# Supplementary Tables

**Supplementary Table 1.** Antibodies used in the study.

| Antibody | **Species** | **Dilution** | **Company, SKU** |
| --- | --- | --- | --- |
| Anti-Pan-cytokeratin | Rabbit | 1:200 | Acris, AM33270RP-N |
| Anti-Claudin-1 | Rabbit | 1:800 | Biorbyt, orb677488 |
| Anti-Acetylated tubulin | Mouse | 1:800 | Sigma-Aldrich, T7451 |
| Anti-FOXJ1 | Rabbit | 1:500 | Invitrogen, PA5-52189 |
| Anti-TP63 | Mouse | 1:50 | Santa Cruz, sc-25268 |
| Anti-CCSP | Rabbit | 1:50 | Invitrogen, PA5-95864 |
| Anti-ACE2 | Goat | 1:20 | RnD Systems, AF933-SP |
| Anti-SARS-CoV-2 Nucleocapsid | mouse | 1:2000 | antibodies-online, ABIN6952435 |
| Anti-pPAK1/2 | Rabbit | 1:100 | CellSignaling, 2601T |
| Anti-Rabbit Alexa 488 | Goat | 1:400 | Invitrogen, A-11008 |
| Anti-Goat Alexa 488 | Donkey | 1:400 | Invitrogen, A-11055 |
| Anti-Mouse Alexa 594 | Goat | 1:400 | Invitrogen, A-11012 |
